# Evidence synthesis in pulmonary arterial hypertension: a systematic review and critical appraisal

**DOI:** 10.1186/s12890-020-01241-4

**Published:** 2020-07-28

**Authors:** Max Schlueter, Amélie Beaudet, Evan Davies, Binu Gurung, Andreas Karabis

**Affiliations:** 1 IQVIA, 210 Pentonville Road, London, N1 9JY UK; 2 Actelion Pharmaceuticals Ltd, Gewerbestrasse 16, CH-4123 Allschwil, Switzerland; 3 IQVIA, Herikerbergweg 314, 1101 CT Amsterdam, Netherlands; 4 Department of Statistical Science, University College London, London, WC1E 6BT UK

**Keywords:** Pulmonary hypertension, Evidence synthesis, Quality appraisal, Literature reviews, Meta-analysis, Network meta-analysis

## Abstract

**Background:**

The clinical landscape of pulmonary arterial hypertension (PAH) has evolved in terms of disease definition and classification, trial designs, available therapies and treatment strategies as well as clinical guidelines. This study critically appraises published evidence synthesis studies, i.e. meta-analyses (MA) and network-meta-analyses (NMA), to better understand their quality, validity and discuss the impact of the findings from these studies on current decision-making in PAH.

**Methods:**

A systematic literature review to identify MA/NMA studies considering approved and available therapies for treatment of PAH was conducted. Embase, Medline and the Cochrane’s Database of Systematic Reviews were searched from database inception to April 22, 2020, supplemented by searches in health technology assessment websites. The International Society for Pharmacoeconomics and Outcomes Research (ISPOR) checklist covering six domains (relevance, credibility, analysis, reporting quality and transparency, interpretation and conflict of interest) was selected for appraisal of the included MA/NMA studies.

**Results:**

Fifty-two full publications (36 MAs, 15 NMAs, and 1 MA/NMA) in PAH met the inclusion criteria. The majority of studies were of low quality, with none of the studies being scored as ‘strong’ across all checklist domains. Key limitations included the lack of a clearly defined, relevant decision problem, shortcomings in assessing and addressing between-study heterogeneity, and an incomplete or misleading interpretation of results.

**Conclusions:**

This is the first critical appraisal of published MA/NMA studies in PAH, suggesting low quality and validity of published evidence synthesis studies in this therapeutic area. Besides the need for direct treatment comparisons assessed in long-term randomized controlled trials, future efforts in evidence synthesis in PAH should improve analysis quality and scrutiny in order to meaningfully address challenges arising from an evolving therapeutic landscape.

## Background

Pulmonary arterial hypertension (PAH) is a rare and debilitating chronic disease of the pulmonary vasculature [1]. Disease progression is characterized by increasing pulmonary vascular resistance (PVR) and non-specific symptoms (e.g., dyspnoea during exercise, fatigue, chest pain, and light-headedness), that ultimately leads to right heart failure and premature death [1, 2]. Prior to the availability of PAH-specific therapies, median survival time was documented as 2.8 years in the US patients with PAH [3]. Five-year survival rate in newly diagnosed patients is reported to be 61.2% [4].

Therapies in PAH have been approved with one or more routes of administration for three key pathogenesis pathways. Approved therapies targeting the nitric oxide pathway are the phosphodiesterase-5 inhibitors (PDE-5I): sildenafil (oral or intravenous [IV]) and tadalafil (oral), and the soluble guanylate cyclase stimulator (sGCS) riociguat (oral). Therapies targeting the endothelin pathway currently approved are macitentan, bosentan and ambrisentan, all administered orally. One of the endothelin receptor antagonist (ERA) drugs, sitaxentan, was authorised in Europe in 2006, but subsequently withdrawn due to liver toxicity [5]. Approved drugs targeting the prostacyclin [PGI2] pathway include epoprostenol (IV), iloprost (inhaled), treprostinil (IV, inhaled, oral, subcutaneous [SC]), beraprost (oral), and selexipag (oral), a selective non-prostanoid PGI2 receptor (IP receptor) agonist.

The treatment of PAH is guided by an evidence-based treatment algorithm published by the European Society of Cardiology and European Respiratory Society (ESC/ERS) [2]. The overall treatment goal is to achieve a low-risk status, associated with World Health Organization (WHO) Functional Class II, and good exercise capacity (> 440 m in the 6-min walking distance test), and right-ventricular function assessed using echocardiography. The latest guidance and proceedings (see Figure S1 in the electronic supplementary material) recommend either monotherapy or initial oral combination therapy for treatment-naïve patients at a low or intermediate risk of clinical worsening or death [2, 6]. For these patients, oral therapies are recommended, therefore ERA and PDE-5I are generally used as first-line treatment. For patients who fail to achieve an adequate clinical response (i.e. a low-risk status after 3 to 6 months) with initial therapy, treatment with sequential double or triple combination therapy is recommended. For high-risk treatment-naïve patients, an initial combination therapy regimen including a drug targeting the PGI2 pathway requiring continuous IV administration is indicated.

A lack of head-to-head treatment comparisons in randomized controlled trials (RCTs) has compounded clinical decision-making in PAH. As a result, a multitude of meta-analyses (MA; the synthesis of evidence from the same treatment comparisons assessed in clinical trials [7]) and network meta-analyses (NMA; the synthesis of evidence from both direct and indirect evidence to allow treatment comparisons that have not been directly assessed in clinical trials [7]) in PAH have been conducted.

Given the absence of direct RCT comparisons and the evolution of disease definition, classification, trials designs, available therapies and treatment guidelines, it is important to better understand the quality of published MA and NMA in PAH and their alignment with clinical decision-making today. The objective of the study was to critically appraise the quality and validity of published MA and NMA studies in PAH and explore the impact of the findings from these studies on current decision-making.

## Methods

### Search strategy and data collection

A systematic literature review was conducted according to the recommendations of the Cochrane Collaboration [8] and the Preferred Reporting Items for Systematic Reviews and Meta-Analyses (PRISMA) guidelines [9], to identify published evidence synthesis (i.e. MA and NMA) studies in PAH.

Searches were conducted from the database inception to September 12, 2018 and updated on April 22, 2020 in Embase, Medline (including Medline-In-Process) and the Cochrane’s Database of Systematic Reviews via OVID in line with The National Institute for Health and Care Excellence (NICE) technology appraisal guidelines and recommendation from Centre for Review and Dissemination and the Cochrane Collaboration [10–12]. Supplementary searches included websites of selected health technology assessment agencies.

Retrieved records were assessed by one reviewer against the pre-specified PICOS criteria (Table S1 in the electronic supplementary material) and unblinded assessments were double checked by the second reviewer. Any discrepancies were resolved through discussion with a third reviewer. Studies were included if they met the following criteria: 1) adult patients with any etiology of PAH (pulmonary hypertension (PH) Group 1) [2], 2) at least two approved and available therapies or drug classes for treatment of PAH (to allow assessment of relative efficacy and safety of compared treatments), 3) full-text MA/NMA report. Details of the search methodology are provided in Tables S2a-h in the electronic supplementary material.

Key baseline characteristics of patients with PAH from the included RCTs were extracted to explore the extent of heterogeneity across the trials.

### Study appraisal

A targeted review of published checklists for evidence synthesis studies was conducted. Checklists published by NICE [13], ISPOR [14], PRISMA [15] and GRADE [16] were identified. Criteria for checklist selection included:

- Domains covered, such as relevance of research question, methods for establishing the evidence base, assessment for internal validity, statistical methods, and reporting of results
- Suitability to present context, including applicability to different forms of evidence synthesis
- Generalizability
- Acceptability and recognition of the checklist

The ISPOR checklist was deemed the most appropriate as it covers all domains listed in the checklist selection criteria, is suited to the study objective and is applicable to different types of evidence synthesis.

Complementary questions were added to the 26-item ISPOR checklist with questions specific to the disease area and/or study objective. These additional questions are marked as such in the study assessment provided in Table S3 in the electronic supplementary material.

The ISPOR checklist provides for a quality grading whereby an overall assessment of ‘strong’, ‘neutral’ or ‘weak’ is given for each of the six domains (i.e. relevance, credibility, analysis, reporting quality & transparency, interpretation, conflict of interest). However, no explicit criteria are provided for scoring each domain. A set of criteria specific to each domain for quality grading was therefore adopted which is described in Table 1. Study appraisals by one reviewer were double checked by a second reviewer.

**Table 1:** Criteria for scoring each domain in the checklist

Domains	Weak	Neutral	Strong
**Relevance**	At least three of the six checklist items suggested study shortcomings, for example omission of relevant therapies in the analysis, omission of relevant outcomes for evidence synthesis, or inclusion of patients outside the target population.	1–2 checklist items were not addressed satisfactorily; no or insufficient justification for a particular analysis approach was provided (e.g. inclusion of oral therapies only without justification).	All checklist items were appropriately addressed.
**Credibility**	Information omitted or insufficient information provided for at least three of the nine checklist items, for examples, omission of key databases in the SLR, omission of a quality assessment of included studies, or lack of identification of imbalances in the distribution of key effect modifiers prior to the analysis.	1–2 checklist items were not addressed satisfactorily, for example, an adequate search strategy but no transparent reporting of the full search strings, or lack of reporting of the results of the quality assessment.	All checklist item were addressed appropriately. The checklist domain ‘credibility’ includes one question only applicable to NMA studies; this question was not considered for the domain grading of MA studies.
**Analysis**	At least three of the 10 checklist items suggested study shortcomings, such as lack of subgroup analyses or meta-regression in cases of between-study heterogeneity, pooling of drug classes, treatments or doses without proper justification, or lack of a valid rationale for the use of random effects or fixed effect models.	1–2 checklist items were not addressed satisfactorily, such as insufficient detail on the statistical model.	All checklist items were addressed appropriately. The checklist domain ‘analysis’ includes four questions only applicable to NMA studies; these questions were not considered for the domain grading of MA studies.
**Reporting quality & transparency**	At least two of the six checklist items were not addressed satisfactorily, or discussion of the impact of important patient characteristics on treatment effects was not included.	Insufficient information for one checklist item or a brief discussion of the impact of the impact of patient characteristics on analysis results was provided.	All checklist items were addressed appropriately. The checklist domain ‘reporting quality & transparency’ includes four questions only applicable to NMA studies; these questions were not considered for the domain grading of MA studies.
**Interpretation**	Results were not contextualized with consideration of limitations or specific treatments were endorsed over others despite a lack of discussion of between-study heterogeneity and/or despite pooling of active therapies.	Study limitations (e.g. between-study heterogeneity) were provided however without a detailed discussion of the impact these may have had on observed study results.	All these aspects were addressed appropriately.
**Conflict of interest**	No information on conflicts of interest was provided, or details of author disclosures and contributions were insufficient.	Disclosures as well as author contributions were clearly stated in cases of personal or financial relationships of affiliations that could have biased the work in question.	No personal or financial relationships or affiliations (that could have biased the study) were declared.

*MA* Meta-analysis, *NMA* Network meta-analysis, *SLR* Systematic literature review

## Results

### Study characteristics

A total of 52 MA and NMA studies met the inclusion criteria and were retained for data extraction and quality appraisal. From electronic database searches, 51 full-text publications were included. From the hand-search of publicly available websites of health technology assessment bodies, one report of the Canadian Agency for Drugs and Technologies in Health was included. The PRISMA diagram in Figure S2a-b (see electronic supplementary material) presents the search results.

The study characteristics of 52 publications included for appraisal are presented in Table 2. The publication year ranged between 2007 [43] and 2020 [39, 41, 48, 67] with most studies published in recent years. MAs were conducted in 35 studies [17, 19, 20, 22, 23, 26–29, 31, 35–41, 43–47, 49–51, 53, 56, 58–60, 63–66, 69], NMAs in 15 studies [18, 21, 24, 25, 30, 32, 33, 42, 48, 52, 54, 57, 61, 62, 67], both NMA and MA in one study [55], and MA and disproportionality analysis in one study [34]. Of 52 studies, 47 evaluated the impact of PAH interventions in patients with PAH and PAH subgroups (based on aetiology, e.g. idiopathic PAH, familial PAH, connective tissue disease-associated PAH). Patients with PH including PAH and non-PAH patients (e.g. PH due to left sided heart disease) were investigated in four studies [20, 34, 43, 44] while patients with PAH were examined alongside other diseases (e.g. heart failure, prostate cancer) in two studies [45, 58].

**Table 2:** Characteristics of evidence synthesis studies

Study ID (author year)	Patient population	Type of evidence synthesis	Number of studies included	Treatments included	Outcomes included	Quality assessment tool (used for included trials)
Avouac 2008 [17]	Patients with PAH (including idiopathic, secondary to CTD or CHD)	MA	10	Oral ERAs (bosentan, sitaxentan) and PDE-5I (oral sildenafil)	6MWD	Jadad scores
Badiani 2016^a^ [18]	Patients with PAH (including associated PAH and IPAH)	NMA	17	Oral ERAs (bosentan, ambrisentan and macitentan), oral PDE-5Is (sildenafil, tadalafil and vardenafil^d^), prostanoids (oral beraprost and oral treprostinil) sPRA (oral selexipag), sGCS (oral riociguat)	Composite clinical worsening	Not reported
Bai 2011 [19]	Patients with PAH	MA	6	Oral PDE-5Is (tadalafil and sildenafil), ERA (bosentan), prostanoids (inhaled iloprost and IV epoprostenol) developed and approved for PAH; combination therapies only included with 2 or 3 drugs.	6MWD, clinical worsening, NYHA FC, mPAP, RAP, PVR and cardiac output, SAEs, all-cause mortality	Quality assessment completed, tool not stated.
Barnes 2019 [20]	Patients with PH (all groups 1–5)	MA	3 (for PAH)	Oral PDE5Is (sildenafil, tadalafil), oral ERAs (ambrisentan, bosentan)	Primary outcomes: WHO FC, 6MWD and mortality. Secondary outcomes: Haemodynamic parameters, quality of life/health status, dyspnoea, clinical worsening (hospitalisation/intervention), and AEs	Cochrane’s risk of bias
Biondi-Zoccai 2013 [21]	Patients with PAH	NMA	6	First line oral drugs: oral ERAs (bosentan, sitaxentan, ambrisentan, prostacyclin analogues (oral beraprost), oral PDE-5Is (tadalafil, sildenafil)	All-cause mortality, clinical improvement and clinical worsening	Cochrane’s risk of bias
Chen 2009 [22]	Patients with PAH and subgroups (i.e. idiopathic PAH, CTD-associated PAH)	MA	20	Any of epoprostenol (IV), iloprost (inhaled), bosentan (oral), sitaxentan (oral) and sildenafil (oral)	Survival, time to clinical deterioration, HRQoL, 6MWD, symptomatic improvement, frequency and duration of hospitalization and outpatient/GP visits, SAEs, AEs, withdrawal, haemodynamic assessment	Quality assessment completed, tool not stated
Coeytaux 2014 [23] [McCrory 2013 full report]	Patients with PAH	NMA	28	Oral PDE-5I (sildenafil and tadalafil), oral ERAs (bosentan and ambrisentan), prostanoids (IV epoprostenol, inhaled iloprost and IV or SC treprostinil) and calcium channel blockers	Mortality, 6MWD, hospitalization, hemodynamic measures (i.e. PVR, PAP, cardiac index), and commonly reported AEs.	Quality appraisal approach as described in the US Agency for Healthcare Research and Quality’s “Methods Guide for Effectiveness and Comparative Effectiveness Reviews.
Dranitsaris 2009 [24]	Patients with PAH	MA	9	Oral treatments: ambrisentan, bosentan, sitaxentan and sildenafil	6MWD, BDI, NYHA Functional Class and clinical worsening	Not reported
Duo-Ji 2017 [25]	Patients with symptomatic PAH, idiopathic PAH or PAH associated with other diseases	NMA	10	Oral ERAs only (ambrisentan, bosentan, sitaxentan and macitentan)	6MWD, clinical worsening, SAE, mortality and all-cause discontinuation	Jadad scores
Fox 2011^b^ [26]	Patients with PAH (including idiopathic PAH, familial PAH, CTD associated PAH, pulmonary shut, portal hypertension, HIV infection and thyroid disease)	MA	6	Oral PDE-5I (sildenafil and tadalafil, ERA (oral bosentan), prostanoids (IV epoprostenol, inhaled iloprost and inhaled treprostinil developed and approved for PAH	6MWD, clinical worsening, mortality, hospitalization for PAH deterioration, lung transplantation, escalation of treatment and safety outcomes	Jadad scores
Fox 2016 [27]	Patients with PAH	MA	18	Prostanoids (IV epoprostenol, inhaled iloprost and inhaled/oral treprostinil) oral ERAs (bosentan, ambrisentan, sitaxsentan and macitentan), oral PDE-5I (sildenafil and tadalafil), sGCS (oral riociguat), sPRA (oral selexipag) with their approved dose	Primary outcomes: all-cause mortality (analysed separately) and composite clinical worsening. Secondary outcomes: 6MWD, PAP, cardiac index, WHO Functional Class.	Cochrane’s risk of bias
Gabler 2012 [28]	Patients with PAH (including idiopathic PAH, CTD-associated PAH, CHD-associated PAH, HIV infection)	MA	10	Oral PDE-5I (sildenafil and tadalafil), oral ERAs (ambrisentan, bosentan and sitaxentan) and prostanoids (inhaled iloprost and SC treprostinil)	6MWD, mortality, lung transplantation, atrial septostomy, hospitalization due to PAH worsening, withdrawal for worsening right-sided heart failure, or addition of other PAH medications	Not reported
Galie 2009b [29]	Patients with PAH	MA	21	Both approved and not approved treatments for PAH (oral ambrisentan, oral bosentan, oral sitaxentan, oral sildenafil, inhaled iloprost, oral beraprost, IV epoprostenol, SC treprostinil, oral terbogrel^d^)	Primary outcome: all-cause mortality Secondary outcomes: PAH-related hospitalizations to PAH, 6MWD, NYHA/ WHO Functional Class, RAP, PAP, cardiac index, and PVR	Not reported
Gao 2017 [30]	Patients with PAH	NMA	32	Prostanoids (IV epoprostenol, inhaled iloprost, oral beraprost and oral/inhaled/SC treprostinil), oral ERAs (bosentan, ambrisentan and macitentan), oral PDE-5Is (sildenafil, tadalafil and vardenafil^d^), sGCS (oral riociguat), and combination therapy regardless of drug dosage forms	Primary endpoint: 6MWD Secondary endpoints: PAP, PVR, all-cause mortality, and composite clinical worsening. Safety endpoint: SAEs	Jadad scores
He 2010 [31]	Patients with PAH	MA	11	Oral bosentan, oral sildenafil and inhaled iloprost	Clinical worsening, NYHA/WHO Functional Class, 6MWD, and hemodynamic parameters including systolic PAP, PAP, PVR, cardiac output and cardiac index, treatment-related SAEs.	Juni scale
Igarashi 2016 [32]	Patients with PAH	NMA	7	5 oral PAH treatments: ambrisentan, bosentan, sildenafil, tadalafil, and beraprost	6MWD, WHO Functional Class and PAP	Cochrane’s risk of bias
Jain 2017 [33]	Patients with symptomatic PAH	NMA	31	All US-FDA approved PAH-specific drugs: oral ERAs (bosentan, ambrisentan and macitentan), oral PDE-5Is (sildenafil and tadalafil), prostanoids (oral/inhaled/SC/IV treprostinil, inhaled iloprost and IV epoprostenol), sGCS (oral riociguat) and sPRA (oral selexipag)	Primary efficacy outcome: composite clinical worsening Secondary efficacy outcomes: PAH-related hospitalization and all-cause mortality Safety outcome: treatment-related AEs leading to drug discontinuation	Cochrane’s risk of bias
Khouri 2018 [34]	Patients with PH in the main analysis; patients with PAH in the sensitivity analysis	MA and a disproportionality analysis	13 (7 in PAH patients)	Oral PDE-5Is (sildenafil and tadalafil) and sGCS (oral riociguat)	AEs	Cochrane’s risk of bias and GRADE for evidence
Kirtania 2019 [35]	Patients with PAH of any aetiology	MA	7	Combination of oral ERAs (ambrisentan, bosentan, macitentan, sitaxentan) with oral PDE5Is (sildenafil or tadalafil), ERA or PDEI monotherapies	Primary outcome: 6MWD Secondary outcomes: Clinical worsening (death, hospitalisation, WHO FC, lung transplantation, clinical deterioration of PAH requiring additional therapy, PVR and NT-proBNP	Cochrane’s risk of bias
Kuwana 2013 [36]	Patients with PAH and CTD-associated PAH	MA	19	Oral PDE-5I (sildenafil and tadalafil), oral ERAs (bosentan and ambrisentan), prostacyclin analogues (IV epoprostenol, oral beraprost, inhaled iloprost and IV/SC/inhaled treprostinil)	6MWD	Cochrane’s risk of bias
Lajoie 2016 [37]	Patients with PAH (including idiopathic PAH, associated PAH, or hereditary PAH)	MA	17	Prostanoids (IV epoprostenol, inhaled/oral treprostinil, inhaled iloprost), oral ERAs (bosentan, ambrisentan and macitentan), oral PDE-5I (sildenafil, tadalafil and vardenafil^d^) or sGCS (oral riociguat)	Primary outcome: clinical worsening Secondary outcomes: all-cause mortality, PAH-related mortality, PAH-related hospitalizations, lung transplantation, treatment escalation, symptomatic progression, WHO Functional Class, exercise capacity, treatment discontinuation, and treatment duration	Cochrane’s risk of bias
Lajoie 2018 [38]	Patients with PAH (including idiopathic PAH and associated PAH)	MA	15	Currently licensed PAH-specific therapies: prostanoids (IV epoprostenol, inhaled iloprost, inhaled/oral treprostinil), oral ERAs (ambrisentan, bosentan, and macitentan), oral PDE-5Is (sildenafil, tadalafil, and vardenafil^d^), sGCS (oral riociguat), and a sPRA (oral selexipag)	Clinical worsening	Cochrane’s risk of bias
Lei 2020 [39]	Patients with CTD-associated PAH or SSc-PAH	MA	27	Combination of oral ERAs (ambrisentan, bosentan) with oral PDE5Is (sildenafil or tadalafil), oral ERA or oral PDEI monotherapies	6MWD, hemodynamics parameters (PVR, PAP) not analysed due to insufficient data	Cochrane’s risk of bias
Li 2013 [40]	Patients with PAH	MA	14	Prostanoids (IV epoprostenol, inhaled iloprost, SC/inhaled treprostinil, oral beraprost)	Efficacy or safety endpoints (e.g. 6MWD, NYHA Functional Class, PAP, PVR, or all-cause mortality)	Jadad scores
Li 2020 [41]	Patients with PAH	NMA	9	Oral ambrisentan, oral bosentan, oral sildenafil	6MWD, PAP, cardiac index, PVR, RAP and mortality	Cochrane’s risk of bias and Jadad score
Lin 2018 [42]	Patients with PAH	NMA	43	Oral ERAs (bosentan, macitentan, sitaxentan and ambrisentan) sGCS (oral riociguat), oral PDE-5Is (sildenafil, tadalafil and vardenafil^d^), Prostanoids (IV epoprostenol, IV/inhaled/oral/SC treprostinil, inhaled iloprost and oral beraprost), and sPRA (oral selexipag) monotherapy or in combination	6MWD, Functional Class amelioration, mortality, clinical worsening, SAEs, withdrawal, PVR, PAP, cardiac index, and RAP	Jadad scores
Liu 2016 [68]	Patients with PAH	MA	35	Prostanoids (IV epoprostenol, inhaled/IV/SC/oral treprostinil, inhaled iloprost, oral beraprost and oral selexipag), oral ERAs (bosentan, ambrisentan and macitentan), oral PDE-5I (sildenafil, tadalafil and vardenafil^d^) sGCSs (oral riociguat) and rho-kinase inhibitor (fasudil^d^; ROA unclear)	Primary outcomes: Mortality, 6MWD, WHO/NYHA Functional Class Secondary outcomes: Cardiopulmonary hemodynamics including PAP, PVR, cardiac index, withdrawal due to AEs	Cochrane’s risk of bias
Macchia 2007 [43]	Patient with PH (including primary PH due to CTD and PH related to thromboembolic disease)	MA	16	Prostanoids (IV epoprostenol, SC treprostinil, inhaled iloprost and oral beraprost), oral ERAs (sitaxentan and bosentan, and PDE-5I (oral sildenafil)	Total mortality, NYHA Functional Class and 6MWD	Not reported
Macchia 2010 [44]	Patients with PH (including idiopathic PAH and PAH-related conditions)	MA	26	Prostanoids (inhaled iloprost, SC treprostinil and IV epoprostenol), oral ERAs (bosentan, ambrisentan and sitaxentan), and oral PDE-5I (sildenafil and tadalafil)	Total mortality, NYHA Functional Class and 6MWD	Not reported
Pan 2017 [45]	Patients with different diseases including PAH	MA	33	All oral ERAs (atrasentan^d^, avosentan^d^, ambrisentan, bosentan, darusentan^d^, macitentan, sitaxentan and zibotentan^d^)	Mortality, CVD increased risk, AEs	The Newcastle–Ottawa scale
Pan 2018 [46]	Patients with CTD-associated PAH only	MA	6	Prostanoids (IV epoprostenol, inhaled treprostinil, and inhaled iloprost), oral ERAs (ambrisentan, bosentan and macitentan), oral PDE-5Is (sildenafil and tadalafil, vardenafil^d^), sGCSs (oral riociguat) and sPRA (oral selexipag)	Primary outcome: composite clinical worsening Secondary outcomes: 6MWD, N-terminal pro-B type natriuretic peptide (NT-proBNP), WHO/NYHA Functional Class or cardiopulmonary hemodynamics	Cochrane’s risk of bias
Paramothayan 2009 [47]	Patients with primary PH and its variant	MA	9	Prostanoids (IV/inhaled Iloprost, IV epoprostenol, IV/SC/oral treprostinil and oral beraprost)	Primary outcomes: 6MWD NYHA Functional Class Secondary outcomes: Mortality and AEs	The Cochrane approach and the Jadad score
Petrovic 2020a [48]	Patients with PAH	NMA	16	Oral ERAs (ambrisentan, bosentan, macitentan), oral PDE5Is (sildenafil, tadalafil), prostanoids (IV epoprostenol, oral/inhaled treprostinil, inhaled iloprost, oral beraprost), sGCSs (oral riociguat), sPRA (oral selexipag) as add-on therapies	6MWD, all-cause mortality, discontinuation due to AEs	Cochrane’s risk of bias
Petrovic 2020b [48]	Patients with PAH	NMA	21	Oral ERAs (ambrisentan, bosentan, macitentan), oral PDE5Is (sildenafil, tadalafil), prostanoids (IV epoprostenol, SC treprostinil, inhaled iloprost, oral beraprost), sGCSs (oral riociguat), sPRA (oral selexipag)	Efficacy outcomes: 6MWD, all-cause mortality Safety outcome: discontinuation due to AEs	Cochrane’s risk of bias
Ryerson 2010 [49]	Patients with PAH	MA	24	Approved prostanoids (IV/inhaled/SC treprostinil, IV epoprostenol and inhaled iloprost oral) ERAs (ambrisentan, bosentan and sitaxentan) and PDE-5I (sildenafil and tadalafil)	Total mortality and other clinical endpoints, including dyspnea, 6MWD, hemodynamics and AEs	The Jadad score and the Cochrane Collaboration’s tool
Savarese 2012 [50]	Patients with PAH	MA	22	Prostanoids (IV epoprostenol, inhaled iloprost, oral beraprost and IV/SC treprostinil), oral ERAs (bosentan, ambrisentan and sitaxentan), oral PDE-5Is (sildenafil, tadalafil and vardenafil^d^) and other drugs (oral imatinib, aspirin; ROA unclear)	Primary endpoint: 6MWD Secondary endpoints: all-cause mortality, hospitalization for PAH and/or lung or heart-lung transplantation, initiation of PAH rescue therapy	Detsky method
Savarese 2013 [51]	Patients with PAH	MA	16	Oral PDE-5I (sildenafil and vardenafil^d^), prostanoids (SC treprostinil, IV epoprostenol and inhaled iloprost), oral ERAs (sitaxentan and bosentan), oral imatinib^d^	Hemodynamic parameters (PAP, PVR, RAP and cardiac index), and clinical events (all-cause mortality, hospitalization for PAH and/or lung or heart-lung transplantation, initiation of PAH rescue therapy)	Detsky method
Silva 2017 [52]	Patients with idiopathic PAH and associated or secondary etiologies (heart failure, CTD-associated, anorexigen use, sickle-cell disease, and HIV)	NMA	20	Prostanoids (IV epoprostenol, SC/oral treprostinil, oral beraprost and inhaled iloprost), oral ERAs (ambrisentan, bosentan and macitentan), oral PDE-5Is (sildenafil, tadalafil and vardenafil^d^), sGCS (oral riociguat)	6MWD, Cardiac index, PAP, PVR, clinical worsening, and mortality	Oxford quality scoring system
Steele 2010 [53]	Patients with idiopathic PAH, or PAH associated with CTD, CHD or HIV	MA	10	Oral bosentan, oral sitaxentan, inhaled iloprost, IV epoprostenol, sildenafil, oral ambrisentan, oral beraprost, inhaled/SC treprostinil, oral tadalafil and oral vardenafil^d^	Primary outcomes: 6MWD Functional Class Secondary outcomes: mortality, AEs	Not reported
Thom 2015 [54]	Patients with PAH	NMA	16 (10 RCTs, 6 observational studies)	Imatinib (oral) as add-on therapy to ERA (oral bosentan), oral PDE-5Is (sildenafil and tadalafil) or prostanoids (IV epoprostenol, inhaled iloprost, inhaled/SC treprostinil and oral beraprost)	6MWD	NICE checklist for RCTs
Tran 2015 [55] [CADTH report]	Patients with PAH	NMA and MA	20	Prostanoids (IV epoprostenol and SC/IV treprostinil), oral ERAs (bosentan, ambrisentan and macitentan), and oral PDE-5Is (sildenafil and tadalafil), sGCS (oral riociguat)	Clinical outcomes: mortality (all-cause, PAH-related), hospitalization, clinical worsening, NYHA/WHO heart failure Functional Class, 6MWD, and BDI and hemodynamic parameters (PVR, PAP, and cardiac index) HRQoL Safety outcomes: AEs, SAEs and treatment discontinuation due to AEs.	A standardized table based on major items from the SIGN 50 instrument. Further critical appraisal performed based on input from clinical experts.
Vizza 2018 [56]	Patients with PAH	MA	6	Oral bosentan, oral ambrisentan, oral riociguat, oral tadalafil and oral/inhaled treprostinil	6MWD	Not reported
Wang 2018^e^ [57]	Patients with PAH	NMA	45	Oral ERAs (ambrisentan, bosentan, macitentan, sitaxsentan), oral PDE5Is (sildenafil, tadalafil, vardenafil^d^), prostanoids (IV epoprostenol, oral/IV/inhaled/SC treprostinil, inhaled iloprost, oral beraprost), sGCSs (oral riociguat), sPRA (oral selexipag)	6MWD, WHO FC, BDI, cardiac index, PAP, RAP, PVR, clinical worsening, hospitalization, death, SAEs, and withdrawal	Not reported
Wei 2016 [58]	Patients with different diseases including PAH	MA	24	Oral ERAs (bosentan, ambrisentan and macitentan); EU authorised	AEs	Cochrane’s risk of bias and GRADE for evidence
Xing 2011 [59]	Patients with PAH (including idiopathic PAH, familial PAH, as well as CTD-associated PAH, pulmonary shut, portal hypertension, HIV infection and thyroid disease)	MA	10	Prostanoids (IV epoprostenol, IV/SC treprostinil, oral beraprost and inhaled iloprost)	6MWD, BDI, cardiac index, mean PAP, PVR, mortality, clinical worsening and AEs	Jadad scores
Zhang 2015 [60]	Patients with PAH	MA	21	Oral treatments (ambrisentan, bosentan, macitentan, sitaxentan, sildenafil, tadalafil, riociguat, beraprost, epoprostenol, treprostinil, terbogrel^d^ and imatinib^d^)	CCW or at least all-cause mortality	Cochrane’s risk of bias
Zhang 2016 [61]	Patients with PAH	NMA	14	Prostanoids (IV epoprostenol, inhaled/IV/oral/SC treprostinil, oral beraprost and inhaled iloprost)	6MWD, mortality, Functional Class, and discontinuation	Not reported
Zhang 2019 [62]	Patients with PAH	NMA	10	Oral ERAs (bosentan, ambrisentan, macitentan)	Safety outcomes: abnormal liver function, peripheral edema and anemia	Cochrane’s risk of bias
Zheng 2014a^c^ [63]	Patients with PAH	MA	18	Oral targeted therapies: prostanoids (beraprost and treprostinil), ERAs (bosentan, ambrisentan and macitentan), PDE-5Is (sildenafil, tadalafil and vardenafil^d^), and sGCS (riociguat)	Primary efficacy outcome: all-cause mortality Secondary efficacy outcomes: clinical worsening, WHO Functional Class, 6MWD Safety outcome: withdrawal due to AEs	Jadad scores
Zheng 2014b [64]	Patients with PAH	MA	14	Prostanoids (IV epoprostenol, inhaled, inhaled/IV/oral/SC treprostinil, inhaled and oral beraprost)	Primary efficacy outcome: all-cause mortality Secondary efficacy outcomes: clinical worsening, 6MWD, and hemodynamic parameters, including PAP, PVR, cardiac index, and mixed venous oxygen saturation. Safety outcome: withdrawal due to AEs	Jadad scores
Zheng 2018 [65]	Patients with PAH	MA	25	Oral prostanoids (treprostinil, beraprost), oral ERAs (ambrisentan, bosentan, macitentan), oral PDE-5Is (sildenafil, tadalafil, vardenafil^d^), sGCSs (oral riociguat), sPRA (oral selexipag)	Primary outcome: composite clinical worsening Secondary outcomes: all-cause mortality, lung transplantation, admission to hospital, treatment escalation, WHO FC improvement, symptomatic progression and 6MWD	Jadad score
Zhu 2012 [66]	Patients with PAH	MA	7	Oral PDE-5Is (sildenafil and tadalafil) oral ERAs (bosentan, sitaxentan and ambrisentan), prostanoids (IV epoprostenol, inhaled iloprost and IV treprostinil)	6MWD, clinical worsening, mortality (data not shown)	Moher 1998 reference provided for the quality assessment

*AEs* Adverse events, *BDI* Borg dyspnea index, *CCW* Combined clinical worsening, *CHD* Congenital heart disease, *CTD* Connective tissue disease, *ERAs* Endothelin Receptor Antagonists, *FC* Functional class, *FPAH* Familial PAH, *HRQOL* Health related quality of life, *IPAH* Idiopathic PAH, *MA* Meta-analysis, *NMA* Network meta-analysis, *NT-proBNP* N-terminal probrain natriuretic peptide, *NYHA/ WHO* New York Heart Association/World Health Organization, *IV* Intravenous, *PAH* Pulmonary arterial hypertension, *PAP* Pulmonary arterial pressure, *PCAs* Prostacyclin analogues, *PDE-5Is* Phosphodiesterase 5 Inhibitors, *PH* Pulmonary hypertension, *PVR* Pulmonary vascular resistance, *RAP* Right atrial pressure, *SAEs* Severe adverse events, *sGCSs* Soluble guanylate cyclase stimulators, *sPRAs* Selective non-prostanoid prostacyclin receptor agonists, *SC* subcutaneous, *SSc-PAH* Pulmonary arterial hypertension related to systemic sclerosis, *6MWD* Six minute walking distance

^a^Although Badiani 2015 reported that prostanoids with IV/inhaled/SC ROA were considered for evaluation, trials on prostanoids with these ROAs were not included in the analysis. No justification provided. ^b^In Fox 2011, sitaxsentan, ambrisentan and vardenafil were included in the search strategy of the review, however, trials with these therapies were not included in the analysis. No justification provided. ^c^ In Zheng 2014a, trials on sitaxentan were excluded from the analysis as it was withdrawn from the market due to liver toxicity. The trial on selexipag was also excluded but provided no justification for the exclusion. ^d^Treatments that have not been approved or made to any markets for adult patients with PAH. ^e^In Wang 2018, a subgroup analysis excluding sitaxsentan was conducted for network comparison of drugs in use on the market

**Fig. 1:**
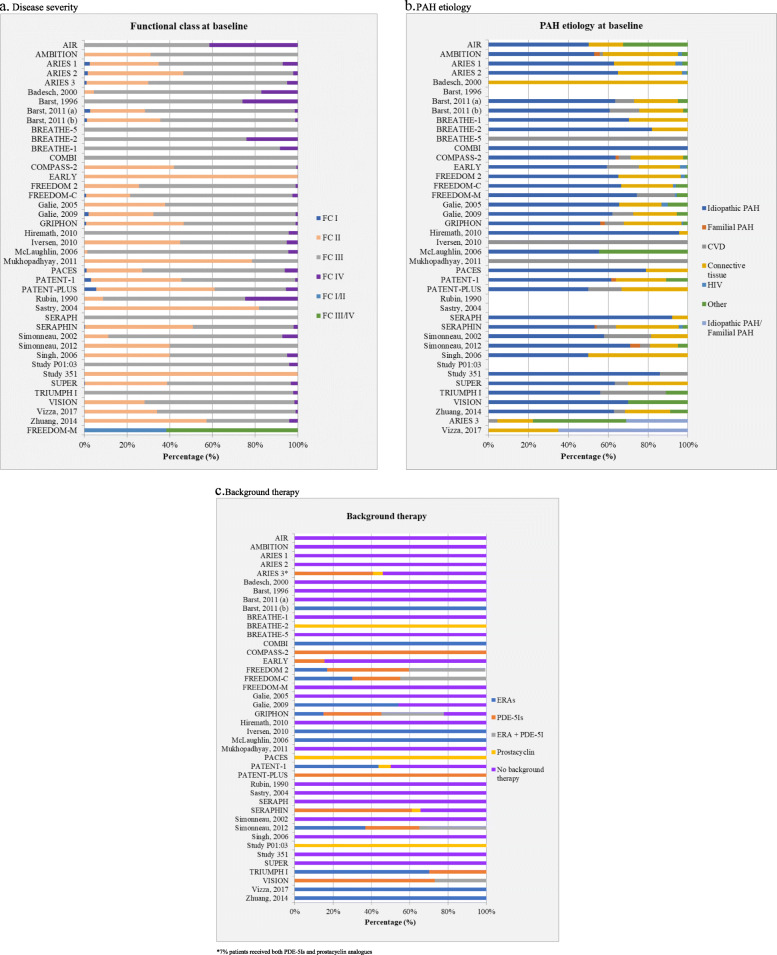
a-c Disease severity, PAH etiology and background therapy across included RCTs

Baseline characteristics of patient populations in the included studies are presented in Fig. 1a-c. The average WHO Functional Class distribution, a measure of disease severity, was 0.6, 30.3, 63.7 and 5.4% for FC I, FC II, FC III and FC IV, respectively.

With a number of exceptions [17, 20, 24, 25, 34, 35, 39–41, 45, 47, 54, 58, 59, 61, 62, 64], most studies investigated treatments targeting all three pathways. All the approved treatments (ERA, PDE-5Is, PRAs, prostacyclin and sGCS) were investigated in nine recent studies [27, 30, 38, 42, 46, 48, 57, 63, 67]. Some studies included treatments approved in limited markets such as beraprost [38, 40, 48, 50, 51, 57, 61, 65, 67]. In nine studies, drugs targeting one pathway only were investigated: prostacyclins in five studies [40, 47, 59, 61, 64] and ERAs in four studies [25, 45, 58, 62]. Fifteen studies [17, 20, 21, 24, 25, 32, 34, 35, 39, 45, 58, 60, 62, 63, 65] focused on oral treatments only. Besides the approved treatments, non-approved PAH treatments were included in seven studies: imatinib [50, 51, 54, 60], terbogrel [29, 60] and aspirin [50]. Despite being withdrawn in 2010, sitaxentan was assessed in four recent studies [25, 35, 57, 60]. Two studies omitted selexipag despite being approved at the time of study [30, 52].

The outcomes evaluated included clinical, hemodynamics, health-related-quality-of -life (HRQoL) and safety. Frequently investigated clinical endpoints were 6MWD (as a standalone or within combined events) in 43 studies [17, 19, 20, 22–33, 35, 36, 39–44, 46–50, 52–61, 63–67] followed by mortality (all-cause or disease-specific) in 37 studies [18–21, 23, 25–30, 33, 37, 40–53, 55, 58–61, 63–67], clinical worsening (standalone or in combined events) in 25 studies [18–21, 24–27, 30, 31, 33, 35, 37, 38, 42, 46, 52, 55, 57, 59, 60, 63–66] and WHO functional class improvement or deterioration in 24 studies [18–20, 24, 27, 29, 31–33, 35, 37, 40, 42–44, 46, 47, 53, 55, 57, 58, 61, 63, 65].

The most commonly employed tool for quality assessment was Cochrane’s risk of bias tool, employed in 21 studies [20, 21, 27, 32–39, 41, 46–49, 58, 60, 62, 67] followed by Jadad scores used in 12 studies [17, 25, 26, 30, 40, 42, 47, 49, 59, 63–65]. There was no mention of quality appraisal being conducted in 10 studies [18, 24, 28, 29, 43, 44, 53, 56, 61, 62].

### Quality appraisal

**Fig. 2:**
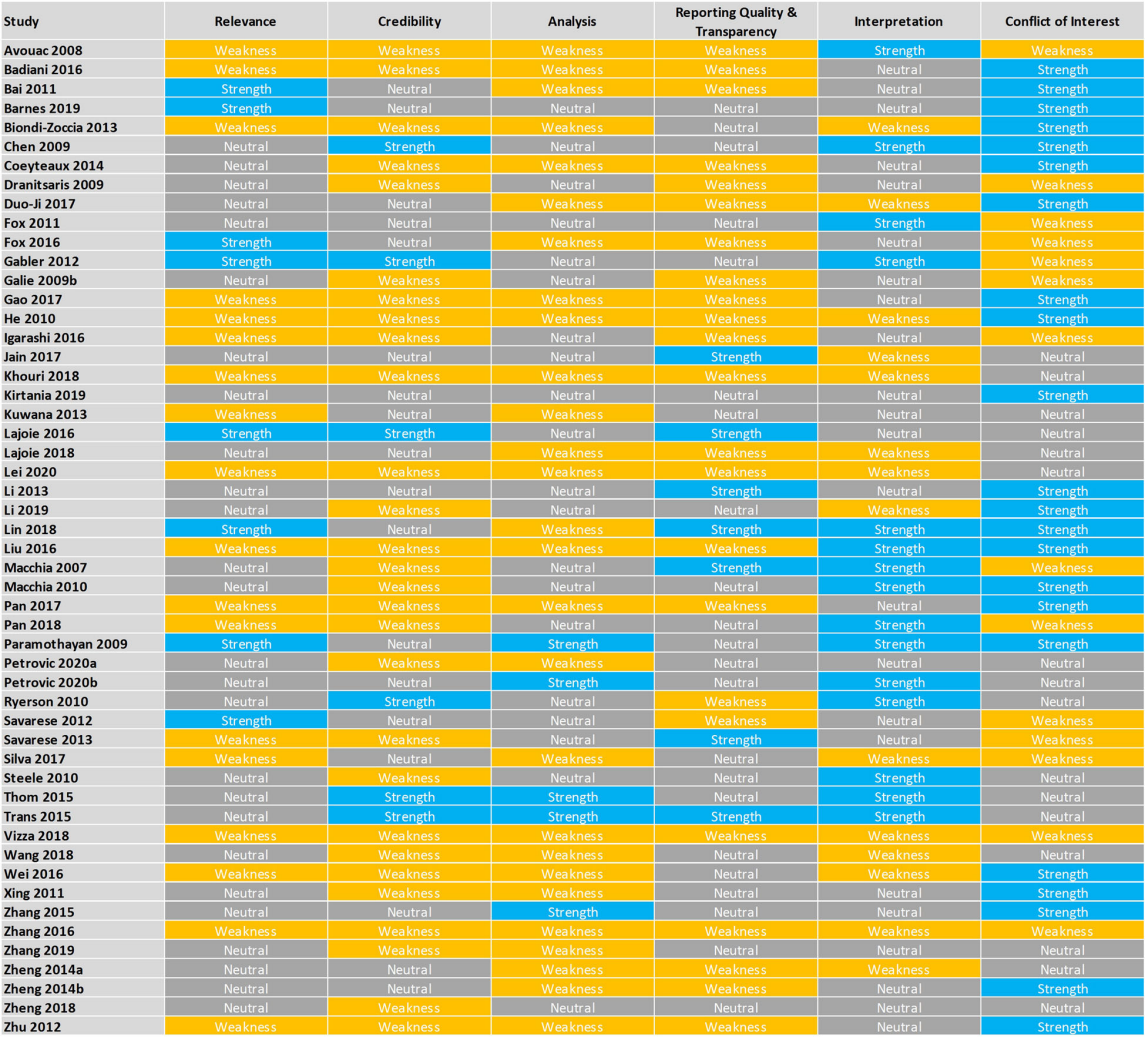
Overview of quality assessment

The quality assessment of the included studies is summarized in Fig. 2 by overall judgement (strength, neutral, weakness) against each domain of the checklist and the number of studies scoring each judgement in each domain in Table 3. The detailed quality assessments are presented in Table S3 in the electronic supplementary material.

**Table 3:** Number of studies with judgement in each domain

Domains of Quality Appraisal	Strength	Neutral	Weakness
Relevance	8	26	18
Credibility	6	18	28
Analysis	5	20	27
Reporting Quality & Transparency	7	22	23
Interpretation	15	23	14
Conflict of Interest	22	16	14

### Relevance

Of the 52 studies reviewed, eight were scored as strong in terms of relevance, 26 as neutral, and the remaining 18 as weak.

Most included studies included relevant populations. In some cases, the population was narrowly defined and thus not generalizable to an overall PAH population (e.g. focused on connective tissue disease-associated-PAH [36, 39]) while in others, it went beyond adult PAH populations (i.e. PH patients [group 2–5] or pediatric PAH were included). Some studies adopted a narrow research focus on 1–2 drug classes [17, 20, 25, 32, 35, 39, 40, 58, 59, 61, 62, 64] or oral therapies only [17, 20, 21, 32, 35, 39, 60, 62, 63, 65], often without explicit and/or adequate justification for such restrictions. Many included studies were highly selective in their choice of outcomes analyzed, 6MWD being the most frequently analyzed outcome.

Very few studies fulfilled the checklist item about the extent to which an evidence synthesis study is informative to decision makers today and aligned with the current clinical practice and guidelines. Several papers did not explicitly state the research question or decision problem guiding the analysis [18, 21, 29, 33, 42, 53, 59]. Several other studies failed to justify the focus or their research question [17, 18, 20, 21, 25, 31, 32, 40, 44, 58, 60–64]. For example, some studies formulated research questions with a very narrow scope (e.g. oral treatments [17, 20, 21, 32, 60, 62, 63]) or included trials with non-PAH populations [34, 43, 44], therefore precluding determination of the optimal choice of therapy based on a comparison of all available treatment options. Some studies included unapproved or withdrawn treatments, while several studies made conclusions at odds with current knowledge, guidelines and clinical practice. For example, claims of PDE-5I monotherapy being superior and a therapy of choice based on older, short-term trials (e.g. Singh 2006 [70], Galie 2005a [71]) are not aligned with evidence from more recent, longer-term studies suggesting that PDE-5I monotherapy is inferior to combination therapy (e.g. SERAPHIN [72], AMBITION [73], GRIPHON [74]). Such inconsistencies across studies challenge a robust interpretation of results for decision makers concerned with a comprehensive assessment of all approved treatments, given the dearth of direct comparisons in RCTs.

### Credibility

Of the 52 studies reviewed, six were scored as strong in terms of credibility, 18 as neutral, and the remaining 28 as weak.

The majority of studies attempted to identify all relevant RCTs. Some studies did not search all of the most relevant databases, i.e. MEDLINE, Embase, CENTRAL [18, 29, 32, 34, 35, 43, 44, 50, 51, 66]. Several studies did not provide details of the search strategy [18–21, 24–26, 29, 31, 32, 35, 36, 39, 40, 43–45, 48, 50–53, 57–59, 61, 65–67] and one study did not provide any details on the search strategy and searched databases [56].

The proposed methodology was found to be relevant to answer the decision problem in almost all included studies. Some studies did not conduct a quality assessment of included RCTs [18, 24, 28, 43, 53, 56, 57]. Several studies did not provide the results of the RCT quality assessment or discuss implications for the analysis in case of poor quality RCTs [21, 23, 30, 31, 36, 39, 45, 60, 61, 66].

Given the absence of randomization across the RCTs included in an MA or NMA, the assessment of effect modifiers is essential to validate assumptions around homogeneity, consistency and transitivity [75, 76]. Effect modifiers are study and patient characteristics associated with treatment effects, capable of modifying (positively or negatively) the observed effect of a risk factor on disease status. Potential effect modifiers in PAH include patient baseline characteristics such as 6MWD, WHO functional class, disease duration, background therapies and etiology; and study design characteristics such as study duration and imputation rules. As the overview of design and patient baseline characteristics of included PAH RCTs (see Fig. 1a-c; Figure S3a-d in the electronic supplementary material) demonstrates, substantial between-study heterogeneity is a feature of every evidence synthesis study in PAH. The majority of studies did not offer a comprehensive assessment prior to analysis or identify imbalances in effect modifiers across the RCTs [17, 18, 20, 21, 23–27, 30, 32, 34, 39, 43–46, 49, 51, 52, 56–64, 66, 67, 69].

### Analysis

Of the 52 studies reviewed, five were scored as strong in terms of analysis, 20 as neutral, and the remaining 27 as weak.

Preservation of study randomization of included RCTs was fulfilled by almost all included studies except in five studies with single-arm [36, 39, 54], retrospective comparative [35] or open-label extension design [56]. Several MAs adopted an approach whereby, for multi-arm trials, the control group was split and the sample size halved [34, 37, 58, 65]. Though outlined in the Cochrane Handbook for Systematic Reviews of Interventions [12], this approach effectively breaks randomization and should therefore be avoided. Other forms of evidence synthesis (e.g. NMA) are more appropriate in this case. Of the included NMA studies with closed loops, most assessed the consistency between the direct and indirect evidence [13, 14, 48, 57, 62].

Common types of analysis to address imbalance in the distribution of treatment effect modifiers include subgroup and sensitivity analysis, meta-regression and using individual patient data. Only about a third of included studies attempted to address between-study heterogeneity [22, 24, 33, 35, 37, 38, 40, 43, 47–51, 54, 55, 59]. The majority of included studies (primarily MAs) used a fixed effects model unless marked heterogeneity was detected (typically assessed using the Cochran Q-test or *I*^*2*^ statistic), in which case a random effects model was used [17, 20, 25, 29, 31, 34, 39, 43, 44, 47, 49, 57, 58, 60, 63–65]. Some studies only fitted a random effects model [19, 20, 23, 26, 27, 35, 40, 45, 46, 48, 62, 67], whereas others only fitted a fixed effects model [28, 30, 38]. The deviance information criterion commonly formed the sole criterion for assessing model fit in the included NMA studies [18, 21, 32] except for Tran et al. 2015 [55], Petrovic 2020a [67] and Petrovic 2020b [48] who assessed model fit based on deviance information criterion and a comparison of the residual deviance with the number of unconstrained data points.

Lastly, several studies pooled treatments at the class level, usually without sound justification for the assumption of a class effect. Very few studies refrained from lumping treatments, doses and co-treatments together [28, 47, 48, 53–55, 60, 62].

### Reporting quality & transparency

Of the 52 studies reviewed, seven were scored as strong in terms of their reporting quality and transparency, 22 as neutral, and the remaining 23 as weak.

All included NMA studies presented a network diagram, except Zhang et al. 2016 [61]. Two of the 11 included NMA studies did not present details of the number and/or RCTs per pairwise comparison [18, 30]. Separate reporting of direct and indirect comparisons was omitted in six NMA studies [18, 25, 30, 48, 54, 67]. A ranking of interventions according to the reported treatment effects was provided by two-third of the included NMA studies [18, 25, 33, 42, 48, 55, 57, 61, 62, 67], some of which did not report associated uncertainty measures. The reporting of all pairwise contrasts between interventions, along with measures of uncertainty, was not adhered by two of the 11 NMA studies [18, 54].

The reporting of individual study results was omitted or not fully reported by 14 of the 52 studies ([21, 25, 30, 32, 38, 42, 45, 48, 53, 55, 57, 61, 62]: Petrovic, 2020a). Overall, 37 of the included studies either completely omitted a discussion or provided a very brief reference to heterogeneity across studies without a specific discussion of the potential impact of differences in patient characteristics on observed results [17–21, 23–27, 29–32, 34–36, 38, 39, 45, 46, 49, 50, 52, 56–63, 65–67].

### Interpretation

Overall, 15 of the 52 studies reviewed were scored as strong in terms of their interpretation of study findings, 23 as neutral, and the remaining 14 as weak.

A number of studies were scored as ‘weak’ when authors did not contextualize results considering limitations [31, 34, 38, 39, 56, 61], or endorsed specific treatments over others without any discussion of between-study heterogeneity and/or despite pooling of active therapies [20, 21, 25, 33, 39, 57, 58]. For example, Jain et al. 2017 [33] combined trials [74, 77, 78] in their primary analysis that differed in patients’ severity level and provision of background therapies.

### Conflict of interest

Among included studies, 22 were scored as strong in terms of conflict of interest,16 as neutral, and the remaining 14 as weak.

Less than a third of all assessed studies provided either no information about conflicts of interest or insufficiently detailed author disclosures. Other studies reported no personal or financial relationships, or clearly stated author contributions in case of personal or final relationships of affiliations that could have biased the respective study.

## Discussion

The objective of this study was to systematically appraise all identified MA/NMA studies in PAH and assess their quality given that such studies are taken into consideration for evidence-based decision-making. To our knowledge, this is the first study of this type in PAH. Overall, the appraisal found most evidence synthesis studies to be of low quality.

Most included evidence syntheses were found not to have defined the decision problem (i.e. the research question underpinning a study), population, selection of comparisons and outcome selection that is compatible or aligned with current clinical practice and treatment guidelines [2, 79]. Of note, the majority of the studies [18–26, 29, 30, 32, 34, 36, 40, 43–47, 49, 52, 53, 55–58, 60–64, 66, 67] included trials that do not reflect today’s clinical practice. For example, the BREATHE-2 [80] and PACES [81] trials investigated bosentan and sildenafil, respectively, as add-on therapy to IV epoprostenol. By contrast, PAH management today typically involves treatment initiation of oral therapy with an ERA and/or PDE-5I in low or intermediate-risk patients comprising the vast majority of patients, whereas parenteral prostacyclins would only be considered or added for high-risk patients [6].

Notably, clinical trial design has evolved from a preponderance of small, short-term and often open-label studies in treatment-naïve patients with severe PAH to larger, longer-term and event-driven trials (such as COMPASS-2 [82], SERAPHIN [72], AMBITION [73], GRIPHON [74]) in largely treatment-experienced and less severe patient populations. Similarly, primary endpoint definition has gradually shifted from improvement in 6MWD to morbidity and mortality as a composite endpoint (with components such as all-cause death, PAH-related hospitalization or disease worsening) which is considered to be a more patient- and clinically relevant endpoint [83–85].

While these changes in trial design and PAH management pose challenges for studies synthesizing evidence generated across such large time spans, a transparent interpretation of findings in recent MA/NMA studies in relation to present clinical practice and guidance was found to be lacking.

A related shortcoming of appraised studies is the choice of outcomes analyzed, which was found to be selective, incomprehensive, and usually not accompanied by clear justification. The most commonly assessed outcome was 6MWD – despite failure of multiple studies to consistently establish significant associations between 6MWD and clinically more relevant outcomes such PAH-related hospitalization, lung transplantation, initiation of rescue therapy or death [28, 29, 43, 50, 86, 87]. Moreover, the assessed evidence synthesis studies generally neither presented a review of the outcome definitions and outcome measures of included trials, nor an assessment of imputation rules for handling missing data.

Mortality was less commonly assessed, which reflects the inherent challenges in designing clinical trials of PAH therapies to detect statistically significant or clinically meaningful differences in mortality. Replication of earlier trials (e.g. Barst 1996 [78]) showing survival benefit over a very short time period and placebo-controlled RCTs comparing monotherapy with no therapy in treatment-naïve patients would be considered unethical today.

Another crucial drawback in most included studies is the lack of a thorough assessment of key effect modifiers prior to the analysis. As the graphs presenting patient baseline characteristics across PAH trials demonstrate (see Fig. 1a-c; Figure S3a-d in the electronic supplementary material), there is marked between-study heterogeneity. One recurring observation was that most evidence synthesis studies included a mix of PAH and non-PAH patients populations, as in the aerosolized iloprost randomized (AIR) study [88] which included PAH and chronic thromboembolic pulmonary hypertension (CTEPH) patients.

Only a handful of studies sought to address such potential systematic differences in the effect modifiers through means of subgroup/sensitivity analyses, meta-regression. This may be due to limited subgroup data available from published PAH RCTs, and challenges around smaller sample sizes associated with subgroup data which results in wider uncertainty estimates and lower likelihood of detecting significant relative treatment effects.

In terms of results synthesis, several studies were found to pool treatments at the drug class level. Best practices guidelines in evidence synthesis, such as NICE DSU TSD 7 [13], recommend against pooling treatment doses or treatments into drug classes since characteristics of the underlying trial population or efficacy/safety trial results may be different.

This review has some limitations. A thorough assessment of the quality of MA/NMA studies is limited by the heterogeneity across included trials. A detailed assessment of between-study heterogeneity in each included MA/NMA was beyond the scope of the review. Nevertheless, a preliminary assessment of patients’ baseline characteristics of all PAH trials included across the appraised MA/NMA studies was considered reflective of most studies. Results or analyses relating to PAH subgroups by etiology, severity or age were not explored further due to no or very limited studies focusing on these specific sub-populations.

## Conclusion

This is the first critical appraisal of published MA/NMA studies in PAH, suggesting overall low quality and validity of efforts synthesizing PAH evidence. As our study demonstrates, this has important implications for clinical decision-making and future research. First, the choice of optimal therapy to maximize patient outcomes should also be guided by a consideration of the limitations of published MA/NMA studies highlighted in this study. Second, future attempts of evidence synthesis in PAH should improve the level of validity and scrutiny to meaningfully address challenges arising from an evolving therapeutic landscape. This should include the definition of decision problems that are aligned with today’s clinical practice and treatment guidelines, justification of key analysis assumptions, a comprehensive interrogation of the evidence base prior to analysis, use of individual patient data to mitigate issues of heterogeneity, and a transparent presentation of results and associated uncertainty measures for all relevant outcomes.

## Supplementary information

**Additional file 1: Table S1.** Eligibility criteria of the Systematic Literature Review. **Table S2a-d.** Search strategies (September 2018). **Table S2e-h.** Search strategies (April 2020 update). **Table S3.** Quality assessment of included evidence synthesis studies. **Figure S1.** Treatment algorithm. **Figure S2a.** PRISMA diagram showing study selection process (September 2018). **Figure S2b.** PRISMA diagram showing study selection process (April 2020 update). **Figure S3a-d.** Mean age, gender, disease duration and 6MWD in included RCTs.

## Data Availability

The datasets analyzed during the current study are available from the corresponding author on reasonable request.

## Abbreviations

CTEPH: Chronic thromboembolic pulmonary hypertension
ERA: Endothelin receptor antagonist
ERS: European Respiratory Society
ESC: The European Society of Cardiology
HRQoL: Health-related-quality-of -life
ISPOR: International Society for Pharmacoeconomics and Outcomes Research
IV: Intravenous
MA: Meta-analyses
NICE: The National Institute for Health and Care Excellence
NMA: Network-meta-analyses
PAH: Pulmonary arterial hypertension
PDE-5I: Phosphodiesterase-5 inhibitors
PICOS: Participants, interventions, comparisons, outcomes, and study design
PH: Pulmonary Hypertension
PRISMA: Preferred Reporting Items for Systematic Reviews and Meta-Analyses
PVR: Pulmonary vascular resistance
RCTs: Randomized controlled trials
sGCS: Soluble guanylate cyclase stimulator
