# Supplementary material for: Evidence synthesis in pulmonary arterial hypertension: a systematic review and critical appraisal
Source: BMC Pulm Med. 2020 Jul 28;20:202. doi: 10.1186/s12890-020-01241-4 (PMC7388228; doi:10.1186/s12890-020-01241-4)
Supplement: Supplementary file 1 — Additional file 1: Table S1. Eligibility criteria of the Systematic Literature Review. Table S2a-d. Search strategies (September 2018). Table S2e-h. Search strategies (April 2020 update). Table S3. Quality assessment of included evidence synthesis studies. Figure S1. Treatment algorithm. Figure S2a. PRISMA diagram showing study selection process (September 2018). Figure S2b. PRISMA diagram showing study selection process (April 2020 update). Figure S3a-d. Mean age, gender, disease duration and 6MWD in included RCTs. [file 12890_2020_1241_MOESM1_ESM.zip › Supplementary_filesR4.docx]

# **Tables**

## ***Data collection***

### Table S1. Eligibility criteria of the Systematic Literature Review

| **PICOS criteria** | **Inclusion criteria** | **Exclusion criteria** |
| --- | --- | --- |
| Population | - Patients with PAH (PH group 1) - Mixed PAH and PH groups 2-5 permitted if PAH subgroups reported | - In vitro studies, - Animal studies, - Paediatric only populations, - Non-PH populations, - PH (groups 2-5) only populations |
| Interventions | - Endothelin receptor antagonist (macitentan, bosentan, ambrisentan), - Phosphodiesterase, pde-5 (sildenafil, tadalafil), - Soluble guanylate cyclase (riociguat), - Prostanoid, prostacyclin (treprostinil, epoprostenol, iloprost, selexipag) - Monotherapy (listed above), - Initial combination therapy, - Sequential combination therapy | - Sitaxsentan only, - Beraprost only, - Iloprost (IV route of administration(ROA)) - No PAH-specific therapies |
| Comparator | All | - |
| Outcomes | All | - |
| Study design | - Meta-analyses comparing at least two active therapies or drug classes* - Network meta-analyses - Indirect treatment comparisons - Articles written in English, French, Spanish or German | - Meta-analyses focusing on one drug or drug class* - Other study types, - Superseded studies (i.e. those with a published update), - Other languages |
|  |  |  |
|  |  |  |

IV, Intravenous; PAH, Pulmonary arterial hypertension; PH: Pulmonary hypertension; ROA: Route of administration

*Meta-analyses with pooling of treatments into drug classes and comparison vs placebo only to be excluded since such analyses preclude treatments comparison that help to inform optimal therapy for PAH

## **Methods**

## ***Data collection***

## Search strategy – September 2018

### Embase search

Database: Embase 1974 to 2018 September 11

Date of search performed:12 September 2018

### Table S2a. Embase search strategy

| **ID** | **Searches** | **Results** |
| --- | --- | --- |
| 1 | exp Hypertension, Pulmonary/ | 78631 |
| 2 | (Pulmonary arterial hypertension or PAH or IPAH or HPAH or FPAH or Primary pulmonary hypertension or PPH or Pulmonary hypertension or Lung arter* hypertension or Lung hypertension).mp. | 105292 |
| 3 | (Pulmonary capillary hemangiomatosis or Pulmonary capillary haemangiomatosis or PCH or Pulmonary veno-occlusive disease or PVOD).mp. | 2069 |
| 4 | (Persistent fetal circulation syndrome or PPHN).mp. | 897 |
| 5 | (Persistent pulmonary hypertension and (newborn or neonat* or infan*)).mp. | 2409 |
| 6 | Eisenmenger complex/ | 2062 |
| 7 | (Eisenmenger* and (disease or syndrome or tetralogy or complex)).mp. | 2381 |
| 8 | or/1-7 | 111286 |
| 9 | Endothelin receptor antagonist/ or Macitentan/ or Bosentan/ or Ambrisentan/ | 12480 |
| 10 | (Endothelin antagonist or Endothelin receptor antagonist* or Endothelin receptor block* or Macitentan or Opsumit or Actelion-1 or "ACT 064992" or ACT064992 or Bosentan or Tracleer or Stayveer or ro470203 or "ro47 0203" or ro 470203 or "ro 47 0203" or Ambrisentan or Letairis or Volibris or bsf208075 or bsf 208075).mp. | 13374 |
| 11 | Phosphodiesterase V inhibitor/ or Sildenafil/ or Tadalafil/ | 25879 |
| 12 | (Phosphodiesterase 5 inhibitor* or Phosphodiesterase type 5 inhibitor* or Phosphodiesterase V inhibitor* or Phosphodiesterase type V inhibitor* or PDE 5 inhibitor* or PDE type 5 inhibitor* or PDE V inhibitor* or PDE type V inhibitor* or PDE5 inhibitor* or Sildenafil or Viagra or Revatio or Acetildenafil or Adonix or Andros or Aphrodil or Desmethylsildenafil or Edegra or Ejertol or Elonza or Emposil or Erectol or Erilin or Eroton or Eroxim or Homosildenafil or Hydroxyhomosildenafil or Mysildecard or Neo Up or NCX-911 or Patrex or Penegra or Granpidam or Rigix or Ripol or Sildefil or Supra or Tigerfil or UK 92480 or UK 92480 or UK 9248010 or Vigain or Vimax or Vizarsin or Xex or Zilden or Zwagra or Tadalafil or Adcirca or Cialis or IC351 or IC 351 or 36 Horas or Forzest or gf 196960 or gf196960 or Pasport or Talmanco or Tardanafil or Xpandyl or Zyalis or Zydalis).mp. | 43901 |
| 13 | Guanylate cyclase/ or Riociguat/ | 11691 |
| 14 | (Guanylate cyclase or Guanylyl* cyclase or Guanosine cyclase or Riociguat or Adempas or bay 63 2521 or bay 632521 or bay632521 or sGC).mp. | 21864 |
| 15 | Prostaglandin/ or Prostanoid/ or Prostacyclin/ or Treprostinil/ or Iloprost/ or Selexipag/ | 72095 |
| 16 | (Prostanoid or Prostacyclin or Cycloprostin or Epoprostenol or Flolan or pgi2 or pgx or Prostaglandin i 2 or Prostaglandin i2 or Prostaglandin x or Caripul or u 53217 or u 53217a or u53217 or u53217a or Veletri or Treprostinil or bw 15au or bw15au or lrx 15 or lrx15 or 15au81 or Remodulin or Tyvaso or orenitram or u 62840 or u62840 or ut 15 or ut 15c or ut15 or ut15c or Iloprost or Ventavis or Uniprost or Ciloprost or Ilomedine or ZK 36374 or ZK36374 or ZK 36375 or ZK36375 or shl401a or sh 401 or sh401 or shl 401a or shl401a or Selexipag or Uptravi or ACT 293987 or ACT293987 or NS 304 or NS-304).mp. | 44411 |
| 17 | ((combination or combined or "add on") adj2 (therap* or treatment*)).mp. | 196007 |
| 18 | or/9-17 | 341591 |
| 19 | (Meta analys#s or Metaanalys#s or NMA or Treatment comparison or ITC).mp. | 245418 |
| 20 | 8 and 18 and 19 | 273 |

### Medline search

Database: Ovid MEDLINE(R) and Epub Ahead of Print, In-Process & Other Non-Indexed Citations, Daily and Versions(R) 1946 to September 11, 2018

Date of search performed:12 September 2018

### Table S2b. Medline search strategy

| **ID** | **Searches** | **Results** |
| --- | --- | --- |
| 1 | exp Hypertension, Pulmonary/ | 32908 |
| 2 | (Pulmonary arterial hypertension or PAH or IPAH or HPAH or FPAH or Primary pulmonary hypertension or PPH or Pulmonary hypertension or Lung arter* hypertension or Lung hypertension).mp. | 58429 |
| 3 | Pulmonary veno-occlusive disease/ | 758 |
| 4 | (Pulmonary capillary hemangiomatosis or Pulmonary capillary haemangiomatosis or PCH or Pulmonary veno-occlusive disease or PVOD).mp. | 2074 |
| 5 | (Persistent fetal circulation syndrome or PPHN).mp. | 1403 |
| 6 | (Persistent pulmonary hypertension and (newborn or neonat* or infan*)).mp. | 1546 |
| 7 | Eisenmenger Complex/ | 1051 |
| 8 | (Eisenmenger* and (disease or syndrome or tetralogy or complex)).mp. | 1477 |
| 9 | or/1-8 | 68608 |
| 10 | exp Endothelin Receptor Antagonists/ | 4716 |
| 11 | (Endothelin antagonist or Endothelin receptor antagonist* or Endothelin receptor block* or Macitentan or Opsumit or Actelion-1 or "ACT 064992" or ACT064992 or Bosentan or Tracleer or Stayveer or ro470203 or "ro47 0203" or ro 470203 or "ro 47 0203" or Ambrisentan or Letairis or Volibris or bsf208075 or bsf 208075).mp. | 6293 |
| 12 | exp Phosphodiesterase 5 Inhibitors/ | 7442 |
| 13 | (Phosphodiesterase 5 inhibitor* or Phosphodiesterase type 5 inhibitor* or Phosphodiesterase V inhibitor* or Phosphodiesterase type V inhibitor* or PDE 5 inhibitor* or PDE type 5 inhibitor* or PDE V inhibitor* or PDE type V inhibitor* or PDE5 inhibitor* or Sildenafil or Viagra or Revatio or Acetildenafil or Adonix or Andros or Aphrodil or Desmethylsildenafil or Edegra or Ejertol or Elonza or Emposil or Erectol or Erilin or Eroton or Eroxim or Homosildenafil or Hydroxyhomosildenafil or Mysildecard or Neo Up or NCX-911 or Patrex or Penegra or Granpidam or Rigix or Ripol or Sildefil or Supra or Tigerfil or UK 92480 or UK 92480 or UK 9248010 or Vigain or Vimax or Vizarsin or Xex or Zilden or Zwagra or Tadalafil or Adcirca or Cialis or IC351 or IC 351 or 36 Horas or Forzest or gf 196960 or gf196960 or Pasport or Talmanco or Tardanafil or Xpandyl or Zyalis or Zydalis).mp. | 24326 |
| 14 | exp Guanylate Cyclase/ | 8592 |
| 15 | (Guanylate cyclase or Guanylyl* cyclase or Guanosine cyclase or Riociguat or Adempas or bay 63 2521 or bay 632521 or bay632521 or sGC).mp. | 16149 |
| 16 | exp Prostaglandins/ | 98014 |
| 17 | (Prostanoid or Prostacyclin or Cycloprostin or Epoprostenol or Flolan or pgi2 or pgx or Prostaglandin i 2 or Prostaglandin i2 or Prostaglandin x or Caripul or u 53217 or u 53217a or u53217 or u53217a or Veletri or Treprostinil or bw 15au or bw15au or lrx 15 or lrx15 or 15au81 or Remodulin or Tyvaso or orenitram or u 62840 or u62840 or ut 15 or ut 15c or ut15 or ut15c or Iloprost or Ventavis or Uniprost or Ciloprost or Ilomedine or ZK 36374 or ZK36374 or ZK 36375 or ZK36375 or shl401a or sh 401 or sh401 or shl 401a or shl401a or Selexipag or Uptravi or ACT 293987 or ACT293987 or NS 304 or NS-304).mp. | 26157 |
| 18 | ((combination or combined or "add on") adj2 (therap* or treatment*)).mp. | 401086 |
| 19 | or/10-18 | 546995 |
| 20 | (Meta analys#s or Metaanalys#s or NMA or Treatment comparison or ITC).mp. | 167518 |
| 21 | 9 and 19 and 20 | 103 |

### Cochrane’s database of systematic review

Database: EBM Reviews - Cochrane Database of Systematic Reviews 2005 to September 5, 2018 
Date of search performed:12 September 2018

### Table S2c. Cochrane search strategy

| **ID** | **Searches** | **Results** |
| --- | --- | --- |
| 1 | (Pulmonary arterial hypertension or PAH or IPAH or HPAH or FPAH or Primary pulmonary hypertension or PPH or Pulmonary hypertension or Lung arter$ hypertension or Lung hypertension).mp. | 208 |
| 2 | (Pulmonary capillary hemangiomatosis or Pulmonary capillary haemangiomatosis or PCH or Pulmonary veno-occlusive disease or PVOD).mp. | 5 |
| 3 | (Persistent fetal circulation syndrome or PPHN).mp. | 14 |
| 4 | (Persistent pulmonary hypertension and (newborn or neonat$ or infan$)).mp. | 37 |
| 5 | (Eisenmenger$ and (disease or syndrome or tetralogy or complex)).mp. | 4 |
| 6 | or/1-5 | 211 |
| 7 | (Endothelin antagonist or Endothelin receptor antagonist$ or Endothelin receptor block$ or Macitentan or Opsumit or Actelion-1 or "ACT 064992" or ACT064992 or Bosentan or Tracleer or Stayveer or ro470203 or "ro47 0203" or ro 470203 or "ro 47 0203" or Ambrisentan or Letairis or Volibris or bsf208075 or bsf 208075).mp. | 21 |
| 8 | (Phosphodiesterase 5 inhibitor$ or Phosphodiesterase type 5 inhibitor$ or Phosphodiesterase V inhibitor$ or Phosphodiesterase type V inhibitor$ or PDE 5 inhibitor$ or PDE type 5 inhibitor$ or PDE V inhibitor$ or PDE type V inhibitor$ or PDE5 inhibitor$ or Sildenafil or Viagra or Revatio or Acetildenafil or Adonix or Andros or Aphrodil or Desmethylsildenafil or Edegra or Ejertol or Elonza or Emposil or Erectol or Erilin or Eroton or Eroxim or Homosildenafil or Hydroxyhomosildenafil or Mysildecard or Neo Up or NCX-911 or Patrex or Penegra or Granpidam or Rigix or Ripol or Sildefil or Supra or Tigerfil or UK 92480 or UK 92480 or UK 9248010 or Vigain or Vimax or Vizarsin or Xex or Zilden or Zwagra or Tadalafil or Adcirca or Cialis or IC351 or IC 351 or 36 Horas or Forzest or gf 196960 or gf196960 or Pasport or Talmanco or Tardanafil or Xpandyl or Zyalis or Zydalis).mp. | 259 |
| 9 | (Guanylate cyclase or Guanylyl$ cyclase or Guanosine cyclase or Riociguat or Adempas or bay 63 2521 or bay 632521 or bay632521 or sGC).mp. | 17 |
| 10 | (Prostanoid or Prostacyclin or Cycloprostin or Epoprostenol or Flolan or pgi2 or pgx or Prostaglandin i 2 or Prostaglandin i2 or Prostaglandin x or Caripul or u 53217 or u 53217a or u53217 or u53217a or Veletri or Treprostinil or bw 15au or bw15au or lrx 15 or lrx15 or 15au81 or Remodulin or Tyvaso or orenitram or u 62840 or u62840 or ut 15 or ut 15c or ut15 or ut15c or Iloprost or Ventavis or Uniprost or Ciloprost or Ilomedine or ZK 36374 or ZK36374 or ZK 36375 or ZK36375 or shl401a or sh 401 or sh401 or shl 401a or shl401a or Selexipag or Uptravi or ACT 293987 or ACT293987 or NS 304 or NS-304).mp. | 124 |
| 11 | ((combination or combined or "add on") adj2 (therap$ or treatment$)).mp. | 2415 |
| 12 | or/7-11 | 2687 |
| 13 | (Meta analys#s or Metaanalys#s or NMA or Treatment comparison or ITC).mp. | 8503 |
| 14 | 6 and 12 and 13 | 68 |

### HTA search strategy

Date of search: 25^th^ October 2018

### Table S2d. HTA bodies search details

| **HTA bodies** | **Search details** | **Publication identified** |
| --- | --- | --- |
| CADTH | Website: <https://www.cadth.ca/>  Search term: Pulmonary arterial hypertension  No of hits: 69 | 1 CADTH report published in 2015 |
| PBAC | Website: <http://www.pbs.gov.au/>  Each specific drug was searched | No reviews identified |

## Search strategy – April 2020 update

### Embase search

Database: Embase 1974 to 2020 April 21

Date of search performed: 22 April 2020

### Table S2e. Embase search strategy (April 2020 update)

| **ID** | **Searches** | **Results** |
| --- | --- | --- |
| 1 | exp Hypertension, Pulmonary/ | 89905 |
| 2 | (Pulmonary arterial hypertension or PAH or IPAH or HPAH or FPAH or Primary pulmonary hypertension or PPH or Pulmonary hypertension or Lung arter* hypertension or Lung hypertension).mp. | 119664 |
| 3 | (Pulmonary capillary hemangiomatosis or Pulmonary capillary haemangiomatosis or PCH or Pulmonary veno-occlusive disease or PVOD).mp. | 2386 |
| 4 | (Persistent fetal circulation syndrome or PPHN).mp. | 1002 |
| 5 | (Persistent pulmonary hypertension and (newborn or neonat* or infan*)).mp. | 2630 |
| 6 | Eisenmenger complex/ | 2265 |
| 7 | (Eisenmenger* and (disease or syndrome or tetralogy or complex)).mp. | 2617 |
| 8 | or/1-7 | 126227 |
| 9 | Endothelin receptor antagonist/ or Macitentan/ or Bosentan/ or Ambrisentan/ | 13535 |
| 10 | (Endothelin antagonist or Endothelin receptor antagonist* or Endothelin receptor block* or Macitentan or Opsumit or Actelion-1 or "ACT 064992" or ACT064992 or Bosentan or Tracleer or Stayveer or ro470203 or "ro47 0203" or ro 470203 or "ro 47 0203" or Ambrisentan or Letairis or Volibris or bsf208075 or bsf 208075).mp. | 14483 |
| 11 | Phosphodiesterase V inhibitor/ or Sildenafil/ or Tadalafil/ | 28524 |
| 12 | (Phosphodiesterase 5 inhibitor* or Phosphodiesterase type 5 inhibitor* or Phosphodiesterase V inhibitor* or Phosphodiesterase type V inhibitor* or PDE 5 inhibitor* or PDE type 5 inhibitor* or PDE V inhibitor* or PDE type V inhibitor* or PDE5 inhibitor* or Sildenafil or Viagra or Revatio or Acetildenafil or Adonix or Andros or Aphrodil or Desmethylsildenafil or Edegra or Ejertol or Elonza or Emposil or Erectol or Erilin or Eroton or Eroxim or Homosildenafil or Hydroxyhomosildenafil or Mysildecard or Neo Up or NCX-911 or Patrex or Penegra or Granpidam or Rigix or Ripol or Sildefil or Supra or Tigerfil or UK 92480 or UK 92480 or UK 9248010 or Vigain or Vimax or Vizarsin or Xex or Zilden or Zwagra or Tadalafil or Adcirca or Cialis or IC351 or IC 351 or 36 Horas or Forzest or gf 196960 or gf196960 or Pasport or Talmanco or Tardanafil or Xpandyl or Zyalis or Zydalis).mp. | 48854 |
| 13 | Guanylate cyclase/ or Riociguat/ | 12579 |
| 14 | (Guanylate cyclase or Guanylyl* cyclase or Guanosine cyclase or Riociguat or Adempas or bay 63 2521 or bay 632521 or bay632521 or sGC).mp. | 23980 |
| 15 | Prostaglandin/ or Prostanoid/ or Prostacyclin/ or Treprostinil/ or Iloprost/ or Selexipag/ | 74611 |
| 16 | (Prostanoid or Prostacyclin or Cycloprostin or Epoprostenol or Flolan or pgi2 or pgx or Prostaglandin i 2 or Prostaglandin i2 or Prostaglandin x or Caripul or u 53217 or u 53217a or u53217 or u53217a or Veletri or Treprostinil or bw 15au or bw15au or lrx 15 or lrx15 or 15au81 or Remodulin or Tyvaso or orenitram or u 62840 or u62840 or ut 15 or ut 15c or ut15 or ut15c or Iloprost or Ventavis or Uniprost or Ciloprost or Ilomedine or ZK 36374 or ZK36374 or ZK 36375 or ZK36375 or shl401a or sh 401 or sh401 or shl 401a or shl401a or Selexipag or Uptravi or ACT 293987 or ACT293987 or NS 304 or NS-304).mp. | 46464 |
| 17 | ((combination or combined or "add on") adj2 (therap* or treatment*)).mp. | 224496 |
| 18 | or/9-17 | 379521 |
| 19 | (Meta analys#s or Metaanalys#s or NMA or Treatment comparison or ITC).mp. | 301455 |
| 20 | 8 and 18 and 19 | 318 |
| 21 | limit 20 to yr="2018 -Current" | 62 |

### Medline search

Database: Ovid MEDLINE(R) and Epub Ahead of Print, In-Process & Other Non-Indexed Citations, Daily and Versions(R) 1946 to April 21, 2020

Date of search performed: 22 April 2020

### Table S2f. Medline search strategy (April 2020 update)

| **ID** | **Searches** | **Results** |
| --- | --- | --- |
| 1 | exp Hypertension, Pulmonary/ | 35691 |
| 2 | (Pulmonary arterial hypertension or PAH or IPAH or HPAH or FPAH or Primary pulmonary hypertension or PPH or Pulmonary hypertension or Lung arter* hypertension or Lung hypertension).mp. | 65015 |
| 3 | Pulmonary veno-occlusive disease/ | 808 |
| 4 | (Pulmonary capillary hemangiomatosis or Pulmonary capillary haemangiomatosis or PCH or Pulmonary veno-occlusive disease or PVOD).mp. | 2261 |
| 5 | (Persistent fetal circulation syndrome or PPHN).mp. | 1501 |
| 6 | (Persistent pulmonary hypertension and (newborn or neonat* or infan*)).mp. | 1646 |
| 7 | Eisenmenger Complex/ | 1089 |
| 8 | (Eisenmenger* and (disease or syndrome or tetralogy or complex)).mp. | 1563 |
| 9 | or/1-8 | 75650 |
| 10 | exp Endothelin Receptor Antagonists/ or Bosentan/ | 5782 |
| 11 | (Endothelin antagonist or Endothelin receptor antagonist* or Endothelin receptor block* or Macitentan or Opsumit or Actelion-1 or "ACT 064992" or ACT064992 or Bosentan or Tracleer or Stayveer or ro470203 or "ro47 0203" or ro 470203 or "ro 47 0203" or Ambrisentan or Letairis or Volibris or bsf208075 or bsf 208075).mp. | 6586 |
| 12 | exp Phosphodiesterase 5 Inhibitors/ or Sildenafil Citrate/ or Tadalafil/ | 8057 |
| 13 | (Phosphodiesterase 5 inhibitor* or Phosphodiesterase type 5 inhibitor* or Phosphodiesterase V inhibitor* or Phosphodiesterase type V inhibitor* or PDE 5 inhibitor* or PDE type 5 inhibitor* or PDE V inhibitor* or PDE type V inhibitor* or PDE5 inhibitor* or Sildenafil or Viagra or Revatio or Acetildenafil or Adonix or Andros or Aphrodil or Desmethylsildenafil or Edegra or Ejertol or Elonza or Emposil or Erectol or Erilin or Eroton or Eroxim or Homosildenafil or Hydroxyhomosildenafil or Mysildecard or Neo Up or NCX-911 or Patrex or Penegra or Granpidam or Rigix or Ripol or Sildefil or Supra or Tigerfil or UK 92480 or UK 92480 or UK 9248010 or Vigain or Vimax or Vizarsin or Xex or Zilden or Zwagra or Tadalafil or Adcirca or Cialis or IC351 or IC 351 or 36 Horas or Forzest or gf 196960 or gf196960 or Pasport or Talmanco or Tardanafil or Xpandyl or Zyalis or Zydalis).mp. | 26604 |
| 14 | exp Guanylate Cyclase/ | 8919 |
| 15 | (Guanylate cyclase or Guanylyl* cyclase or Guanosine cyclase or Riociguat or Adempas or bay 63 2521 or bay 632521 or bay632521 or sGC).mp. | 17114 |
| 16 | exp Prostaglandins/ | 100020 |
| 17 | (Prostanoid or Prostacyclin or Cycloprostin or Epoprostenol or Flolan or pgi2 or pgx or Prostaglandin i 2 or Prostaglandin i2 or Prostaglandin x or Caripul or u 53217 or u 53217a or u53217 or u53217a or Veletri or Treprostinil or bw 15au or bw15au or lrx 15 or lrx15 or 15au81 or Remodulin or Tyvaso or orenitram or u 62840 or u62840 or ut 15 or ut 15c or ut15 or ut15c or Iloprost or Ventavis or Uniprost or Ciloprost or Ilomedine or ZK 36374 or ZK36374 or ZK 36375 or ZK36375 or shl401a or sh 401 or sh401 or shl 401a or shl401a or Selexipag or Uptravi or ACT 293987 or ACT293987 or NS 304 or NS-304).mp. | 26885 |
| 18 | ((combination or combined or "add on") adj2 (therap* or treatment*)).mp. | 428044 |
| 19 | or/10-18 | 579551 |
| 20 | (Meta analys#s or Metaanalys#s or NMA or Treatment comparison or ITC).mp. | 206123 |
| 21 | 9 and 19 and 20 | 127 |
| 22 | limit 21 to yr="2018 -Current" | 35 |

### Cochrane’s database of systematic review

Database: EBM Reviews - Cochrane Database of Systematic Reviews 2005 to April 17, 2020
Date of search performed: 22 April 2020

### Table S2g. CDSR search strategy (April 2020 update)

| **ID** | **Searches** | **Results** |
| --- | --- | --- |
| 1 | (Pulmonary arterial hypertension or PAH or IPAH or HPAH or FPAH or Primary pulmonary hypertension or PPH or Pulmonary hypertension or Lung arter$ hypertension or Lung hypertension).mp. | 230 |
| 2 | (Pulmonary capillary hemangiomatosis or Pulmonary capillary haemangiomatosis or PCH or Pulmonary veno-occlusive disease or PVOD).mp. | 5 |
| 3 | (Persistent fetal circulation syndrome or PPHN).mp. | 16 |
| 4 | (Persistent pulmonary hypertension and (newborn or neonat$ or infan$)).mp. | 42 |
| 5 | (Eisenmenger$ and (disease or syndrome or tetralogy or complex)).mp. | 5 |
| 6 | or/1-5 | 233 |
| 7 | (Endothelin antagonist or Endothelin receptor antagonist$ or Endothelin receptor block$ or Macitentan or Opsumit or Actelion-1 or "ACT 064992" or ACT064992 or Bosentan or Tracleer or Stayveer or ro470203 or "ro47 0203" or ro 470203 or "ro 47 0203" or Ambrisentan or Letairis or Volibris or bsf208075 or bsf 208075).mp. | 22 |
| 8 | (Phosphodiesterase 5 inhibitor$ or Phosphodiesterase type 5 inhibitor$ or Phosphodiesterase V inhibitor$ or Phosphodiesterase type V inhibitor$ or PDE 5 inhibitor$ or PDE type 5 inhibitor$ or PDE V inhibitor$ or PDE type V inhibitor$ or PDE5 inhibitor$ or Sildenafil or Viagra or Revatio or Acetildenafil or Adonix or Andros or Aphrodil or Desmethylsildenafil or Edegra or Ejertol or Elonza or Emposil or Erectol or Erilin or Eroton or Eroxim or Homosildenafil or Hydroxyhomosildenafil or Mysildecard or Neo Up or NCX-911 or Patrex or Penegra or Granpidam or Rigix or Ripol or Sildefil or Supra or Tigerfil or UK 92480 or UK 92480 or UK 9248010 or Vigain or Vimax or Vizarsin or Xex or Zilden or Zwagra or Tadalafil or Adcirca or Cialis or IC351 or IC 351 or 36 Horas or Forzest or gf 196960 or gf196960 or Pasport or Talmanco or Tardanafil or Xpandyl or Zyalis or Zydalis).mp. | 153 |
| 9 | (Guanylate cyclase or Guanylyl$ cyclase or Guanosine cyclase or Riociguat or Adempas or bay 63 2521 or bay 632521 or bay632521 or sGC).mp. | 17 |
| 10 | (Prostanoid or Prostacyclin or Cycloprostin or Epoprostenol or Flolan or pgi2 or pgx or Prostaglandin i 2 or Prostaglandin i2 or Prostaglandin x or Caripul or u 53217 or u 53217a or u53217 or u53217a or Veletri or Treprostinil or bw 15au or bw15au or lrx 15 or lrx15 or 15au81 or Remodulin or Tyvaso or orenitram or u 62840 or u62840 or ut 15 or ut 15c or ut15 or ut15c or Iloprost or Ventavis or Uniprost or Ciloprost or Ilomedine or ZK 36374 or ZK36374 or ZK 36375 or ZK36375 or shl401a or sh 401 or sh401 or shl 401a or shl401a or Selexipag or Uptravi or ACT 293987 or ACT293987 or NS 304 or NS-304).mp. | 126 |
| 11 | ((combination or combined or "add on") adj2 (therap$ or treatment$)).mp. | 1845 |
| 12 | or/7-11 | 2071 |
| 13 | (Meta analys#s or Metaanalys#s or NMA or Treatment comparison or ITC).mp. | 8837 |
| 14 | 6 and 12 and 13 | 64 |
| 15 | limit 14 to yr="2018 -Current" | 29 |

### HTA search strategy

Date of search: 22^nd^ April 2020

### Table S2h. HTA bodies search details

| **HTA bodies** | **Search details** | **Publication identified** |
| --- | --- | --- |
| CADTH | Website: <https://www.cadth.ca/>  Search term: Pulmonary arterial hypertension  No of hits: 72 | No new reviews identified |
| PBAC | Website: <http://www.pbs.gov.au/>  Each specific drug was searched | No new reviews identified |

## Method

## *Study appraisal*

### Table S3. Quality assessment of included evidence synthesis studies

# **Figures**

## Background

### Figure S1. Treatment algorithm

Low or

Maximal medical therapy^i^ and listing for lung transplantation^j^

After 3-6 months of treatment

Intermediate or

high risk^d^

After 3-6 months of treatment

Structured follow-up^g^

]#

Intermediate or

Triple sequential combination^h^

Low risk^d^

]#

Non-vasoreactive

CCB therapy^c^

High risk^d^

Consider referral for lung transplantation

Initial oral combination^f^

Residual role for initial monotherapy [table 2]^e^

Initial combination including IV PCA^f^

intermediate risk^d^

high risk^d^

General measures^a^ Support therapy^b^

Vasoreative

PAH confirmed by expert center

Acute vasoreactivity test IPAH/HPAH/DPAH only

Source: Galie 2018

Abbreviations: PAH, pulmonary arterial hypertension; IPAH, idiopathic PAH; HPAH, heritable

PAH; DPAH, drug-induced PAH; CCB, calcium channel blocker; PCA, prostacyclin analogue; PH, pulmonary hypertension.

^a^2015 ESC/ERS PH guidelines Table 16; ^b^2015 ESC/ERS PH guidelines Table 17; ^c^2015 ESC/ ERS PH guidelines Table 18; ^d^2015 ESC/ERS PH guidelines Table 13; ^e^2015 ESC/ERS PH guidelines Table 19; ^f^2015 ESC/ERS PH guidelines Table 20; ^g^2015 ESC/ERS PH guidelines Table 14; ^h^2015 ESC/ERS PH guidelines Table 21; ^i^maximal medical therapy is considered triple combination therapy including a SC. or an IV PCA (IV preferred in high-risk status); ^j^2015 ESC/ERS PH guidelines Table 22.

## Results

## Study characteristics

### Figure S2a. PRISMA diagram showing study selection process (September 2018)

**Studies included for quality appraisal (n=43)**

Records excluded (n=290)

**Records after duplicates removed and screened**

**(n=363)**

**Records identified through electronic search**

**(n=444)**

Embase (n=273)

Medline (n=103)

CDSR (n=68)

**Full text assessed for eligibility**

**(n=73)**

Full publication (n=63)

Conference abstract (n=10)

**Studies included (n=42)**

Full text articles excluded (n=31)

Study design (n=15)

Intervention (n=1)

Outcomes (n=4)

Superseded publication (n=2)

Conference abstract (n=9)

Articles from other sources (n= 1)

HTA bodies search (n=1)

### Figure S2b. PRISMA diagram showing study selection process (April 2020 update)

**Studies included for quality appraisal (n=9)**

Records excluded (n=79)

**Records after duplicates removed and screened**

**(n=94)**

**Records identified through electronic search**

**(n=126)**

Embase (n=62)

Medline (n=35)

CDSR (n=29)

**Full text assessed for eligibility**

**(n=15)**

Full publication (n=14)

Conference abstract (n=1)

**Studies included (n=9)**

Full text articles excluded (n=6)

Study design (n=1)

Intervention (n=4)

Conference abstract (n=1)

Articles from other sources (n= 0)

HTA bodies search (n=0)

## **Methods**

## ***Data collection***

## Figure S3a-d. Mean age, gender, disease duration and 6MWD in included RCTs

Figure S3a. Mean age

* Iversen 2010 reported median age **Sastry 2004 reported age range

Figure S3b. Gender

Figure S3c. Mean disease duration

* AMBITION reported a mean disease duration of less than one month** Mean disease duration was reported in a format that did not allow it to be included in the graph, however, the majority of patients had a disease duration at baseline of 0-2 years.

Figure S3d. Mean 6MWD at baseline

*Proportion of patients with baseline 6MWD ≥320m reported **6MWD was performed as close to trough levels of sildenafil and peak levels of the bosentan as possible.
